# Orthotropic reconstruction and characterization of bone tissues through a visco-plasto-damage model

**DOI:** 10.1371/journal.pone.0335948

**Published:** 2025-11-07

**Authors:** Gaia Marangon, Riccardo Fincato, Emanuele Luigi Carniel, Ilaria Toniolo, Alice Berardo, Gianluca Mazzucco

**Affiliations:** 1 Department of Mathematics, University of Padova, Padova, Italy; 2 Department of Civil, Environmental and Architectural Engineering, University of Padova, Padova, Italy; 3 Department of Industrial Engineering, University of Padova, Padova, Italy; UNSW: University of New South Wales, AUSTRALIA

## Abstract

This study presents a robust numerical framework for modeling bone tissue mechanics, integrating an orthotropic visco-plasto-damage model with high-resolution bone geometries derived from computed tomography (CT) scans. The model, implemented within a finite element environment, captures essential bone characteristics, including orthotropic elasticity, viscoplasticity, and progressive damage accumulation. To enhance computational efficiency, an octree-based algorithm has been employed to assign local orthotropic axes based on CT data to enable accurate representation of bone mechanical response across complex geometries. Model calibration, based on experimental data from the literature, supported reliable simulations of cortical and trabecular bone, with validation across a range of loading conditions. The practical efficacy of this approach has been demonstrated through a dental implant case study, wherein stress relaxation, plastic deformation, and damage progression within bone tissue were analyzed. Results indicated a pronounced influence of accurate material orientation on the predicted stress distributions, underscoring the necessity of precise orientation for valid biomechanical simulations. The proposed modeling framework may significantly advance the simulation of bone tissue under realistic physiological conditions, with applications in implant design and evaluation. This method provides a scalable solution for simulating orthotropic materials in biomechanical contexts, combining high-fidelity geometrical reconstruction with a suitable constitutive model, thereby offering a valuable tool for the development and optimization of biomedical implants.

## Introduction

Many efforts have been addressed in recent decades with the ambitious goal of defining a complete and versatile constitutive model for bone tissue, capable of representing the wide range of mechanical behaviors displayed by this living material. Indeed, bone shows a complex hierarchical inner structure adding mechanisms at various scales [1] and exhibits material properties, which vary consistently with age, gender, location and function of the bony structure in the organism. These characteristics complicate the task of constructing a unique model capable of adapting to all possible situations, since the constitutive formulation should be sufficiently manageable to be used in a FE code implementation, for predictive simulations in many applications.

Despite this high variability in bone mechanical behavior, some common features can be outlined. They include an initial elastic phase, of orthotropic or transverse isotropic nature (see [2–4]); a subsequent plastic evolution, whose onset - similarly to the elastic properties - is often successfully linked to the microstructure [5–7]; hardening effects of both isotropic [7] and kinematic nature [8], possibly followed by softening effects [9]; progressive damage accumulation, induced by plastic deformations [1,10] and modeled by specific isotropic [7,8] or orthotropic functions [11,12]; viscous effects coupled to elastic or plastic behaviors [13,14]; and eventually recovery phenomena, modeling bone’s self-repair ability [15].

The inclusion of all or few of these cited behaviors must avoid excessive loss of performance in terms of computational time, memory usage and degree of automation, and, at the same time, ensure a reliable description of bone mechanical response. This is essential for application capabilities, allowing to reproduce problems and predict results, even when dealing with complex geometries or other configurations involving large amounts of data.

A typical example is that of CT scans of whole bones, which are frequently used for accurate reconstructions of bone-specific *in-vitro* or *in-vivo* macroscopic tests, but require large amounts of data to be manipulated. In these cases, detailed geometries are generally associated to simpler constitutive models, often discarding plastic, viscous or damage effects in favor of pure elasticity [16–19], while on the other hand, validation of complex constitutive models, covering a wider range of bone mechanical behaviors, is often performed on simpler geometries, such as dumbbell specimens or schematic representations of whole bones [11,20].

However, it is well known that heterogeneity plays a critical role in governing bone mechanical behavior. For example, the work associated with [21] demonstrates how anisotropic, inhomogeneous, orthotropic material properties derived from CT-based or micromechanics-informed data improve finite element model predictions of bone strength and deformation, particularly in patient-specific applications. Such models allow the elastic moduli and yield criteria to vary spatially in accordance with bone density, orientation, and microstructure. Similarly, [22] emphasizes that bone internal heterogeneity—such as variations in mineralization, porosity, and microarchitecture—leads to significant local differences in stiffness, strength, and failure behavior, which simple homogeneous or phenomenological material laws cannot completely describe. Integrating micromechanical or multi-scale constitutive frameworks that explicitly account for heterogeneity (e.g. spatially varying elastic moduli, damage thresholds tied to local microstructure as well as the correct trabecular organization) could therefore potentially improve the accuracy of predictions, especially in regions of high stress concentration or in evaluating failure risk.

The aim of this article is to refine the procedures through which a constitutive model is applied to non-trivial sets of data, giving the possibility to combine the predictive power of a sophisticated mechanical representation with the accuracy in geometrical reconstruction and the inclusion of microstructural heterogeneity obtained by CT scans, thus maintaining, at the same time, an acceptable quality in terms of computational time, memory usage and degree of automation.

To this aim, the constitutive model presented by [8] has been selected as a complete and reliable representation of bone’s mechanical features, involving several of the main properties mentioned above - orthotropic elasticity, viscoplasticity and damage - and exhibiting a considerable number of validations from various authors [23–25]. Following [8], the model was implemented and embedded in a UMAT subroutine, to be used in Abaqus [26] to perform complete mechanical simulations. Prior to testing it on complex geometries, the model was calibrated, selecting plausible values for the material parameters. The calibration was performed both on cortical and trabecular experimental data on bone taken from literature, with the purpose of verifying its versatility and suitability in representing both trabecular and cortical bone.

Moreover, in order to test non-trivial geometries, CT scans of two bones were considered, namely a mandible and a femur, which allowed for an accurate geometrical reconstruction and, at the same time, provided the orthotropic axes of bone at every point of the geometry. Thanks to this local information, the constitutive model was applied, combining the orientation of material properties to the geometrical specificity of the sample under analysis. To maintain efficiency and avoid considerable losses of computational time and memory, this procedure was performed by employing an octree data structure, allowing for an optimized search of the best possible match. Actual assignment was then performed by Abaqus by means of a suitably user-defined ORIENT subroutine. The effect of an increasingly refined assignment of material orientation was then inquired in a set of sensitivity tests on both femur and mandible, qualitatively and quantitatively showing the growing accuracy of the simulations.

After all these validation tests, this work focused attention on a practical application concerning dental implant simulation, thus exploiting the entire procedure of scanning, assigning material orientation and applying the constitutive model to this specific example. The results, which included the prediction of plastic peaks and viscoplastic effects, demonstrated the versatility and accuracy of the presented procedure.

## Materials and methods

### Visco-plasto-damage model

According to the model defined by [8], bone’s first response to external stresses is of elastic type, characterized by an orthotropic or transversely isotropic nature. A stress threshold, defined by means of a yield function Y(σ,κ), determines the transition from elastic to plastic regime, in which unrecoverable strains begin to accumulate. Such process being dependent on the rate of load application, this second phase of bone’s behavior is coupled to viscous effects, modeled through Perzyna formulation [27,28]. Moreover, accumulation of unrecoverable strains is physically related to the occurrence of microcracks, which are modeled by an isotropic damage function acting on the stiffness to progressively reduce the elastic properties of the material.

This mechanical behavior is formalized in the following set of constitutive equations, describing respectively the damaged-elastic evolution, the viscoplastic evolution, and the yield point between the two.

$$\sigma =(1-D(\kappa ))𝕊(\varepsilon -\varepsilon^{p})$$ (1)

$${\dot{\varepsilon }}^{p}=\gamma \;\nabla_{S}Y(\sigma ,\kappa )$$ (2)

$$\bar{Y}(\sigma ,\kappa ,\gamma ):=Y(\sigma ,\kappa )-\phi (\gamma )=0$$ (3)

Firstly, the stress-strain constitutive equation (Eq 1) relates the elastic strains to the corresponding stresses and is defined by means of an orthotropic stiffness tensor, which can be reduced to transverse isotropic by a proper choice of the elastic constants. This tensor is affected by an isotropic damage function as in Eq 4, which depends on the accumulated plastic strains defined by Eq 5 and is ruled by two material parameters *k*_0_ and *k*_*p*_, describing respectively the threshold for damage activation and the rate of its evolution. The former was not considered in the original model by [8], but has been added to account for possible experimental delays between the onset of plastic strains and that of microcracks.

$$D(\kappa )=\left\{ \begin{array}{cc} 0 & \kappa <k_{0}\\ 1-e^{-k_{p}(\kappa -k_{0})} & \kappa \geq k_{0} \end{array}\right.$$ (4)

$$\kappa =\int_{0}^{t}|{\dot{\varepsilon }}^{p}|d\tau$$ (5)

A scalar damage evolution law was adopted instead of an anisotropic one, as this approach enables reliable parameters identification from standard uniaxial tests, while avoiding the large number of parameters typically required by non-scalar formulations, thus ensuring tractability and robustness of the model calibration. Secondly, the plastic flow rule described by Eq 2 is of associated type and, according to Perzyna formulation, is coupled with viscous effects by means of the viscoplastic consistency parameter defined in Eq 6, which is an increasing function of the yield Y(σ,κ) and depends on two material parameters, *m* and *η*.

$$\gamma =\frac{Y^{2}+mY}{\eta }$$ (6)

Finally, the yield surface bounding the elastic region is defined by means of a yield function as in Eq 7, which evolves as plastic strains accumulate and is therefore modeled involving both kinematic and isotropic hardening:

$$Y(\sigma ,\kappa )=\sqrt{\left(\sigma -r(\kappa )A):𝔸(\sigma -r(\kappa )A\right)}-r(\kappa )$$ (7)

Based on an equivalent orthotropic Tsai-Wu formulation, kinematic hardening is introduced by means of a second order tensor A and a fourth order tensor 𝔸, whose definitions involve three vectorial parameters $m_{1}$, $m_{2}$, $m_{3}$ related to fabric and three scalar parameters $\epsilon_{0}^{+}$, $\epsilon_{0}^{-}$, $\xi_{0}$ representing respectively the yield strain in uniaxial tension and compression and the strain interaction coefficient (see the original article by [8] for complete description).

As for the isotropic hardening, in the original model the hardening function r(κ) is described as a monotonic function (Eq 8) of the accumulated plastic strains, involving two material parameters *y*_*r*_ and *s*_*h*_:

$$r^{orig}(\kappa )=1+(y_{r}-1)(1-e^{-s_{h}\kappa })$$ (8)

Even though properly describing the initial expansion of the elastic domain occurring in plasticity, this so-defined function does not catch the subsequent softening phase, particularly visible in experimental compression tests. In order to overcome this deficiency, the function r(κ) was modified as in Eq 9, adding a new decreasing behavior that activates at a threshold level *k*_*s*_ of accumulated plastic strains and which evolves according to material parameter *s*_*s*_. Further improvements of softening formulation, such as coupling it with damage evolution, are out of the scope of this paper.

$$r(\kappa )=\left\{ \begin{array}{cc} 1+(y_{r}-1)(1-e^{-s_{h}\kappa }) & \;\kappa \leq k_{s}\\ r(k_{s})\;e^{-s_{s}\;(\kappa -k_{s})} & \;\kappa >k_{s} \end{array}\right.$$ (9)

Due to viscous effects, the resulting yield function (Eq 7) must always satisfy Eq 6, which can therefore be inverted as in Eq 10 to define a so-called generalized yield function (Eq 3), whose level zero set represents the yield surface bounding the elastic region.

$$Y=-\frac{m}{2}+\sqrt{\frac{m^{2}}{4}+\eta \;\gamma }=:\phi (\gamma )$$ (10)

As a consequence, the generalized yield function (Eq 3) can be used to write the generalized Kuhn-Tucker conditions (Eq 11) and the consistency condition (Eq 12), recovering the standard setting.

$$\bar{Y}\leq 0\;,\;\gamma \geq 0\;,\;\gamma \bar{Y}=0$$ (11)

$$\dot{\bar{Y}}=0$$ (12)

### Model implementation

Since the implementation of the selected constitutive model has already been extensively described by [8], in the following, the return mapping procedure is outlined and described, rearranged in a more general form to facilitate possible modifications.

This part of the implementation concerns the plastic case and allows finding the correct value of the internal variables by constructing and solving the following set of equations:


$$\left\{ \begin{array}{c} \bar{Y}(\sigma ,\gamma ,\kappa )=0\\ R^{\left(\varepsilon \right)}(\sigma ,\gamma ,\kappa )=\frac{𝔼}{1-D(\kappa_{0})}\sigma^{t}-\frac{𝔼}{1-D(\kappa )}\sigma -△t\;\gamma \;\nabla_{S}Y=0\\ h(\sigma ,\gamma ,\kappa )=\kappa_{0}-\kappa +△t\;\gamma |\nabla_{S}Y(\sigma ,\kappa )|=0 \end{array}\right.$$

representing, respectively: the generalized yield surface, already defined in Eq 3; the residual error on the stresses, built after the elastic trial; and the evolution equation of the accumulated plastic strain, expressed as the level zero of a function.

It is most convenient to rewrite the system in vectorial form:


$$b(x)=0\;\;\text{with:}\;x:=[\sigma ,\gamma ,\kappa {]}^{T},\;b(x):=[\bar{Y}(x),R^{\left(\varepsilon \right)}(x),h(x){]}^{T}$$

In this way, the Newton-Raphson procedure can be applied. The vectorial function is expanded and truncated at the first order:

$$b(x)\;≃\;b(x_{0})+B(x-x_{0})=0$$ (13)

with B the jacobian of the vectorial function:

$$B={[\begin{array}{ccc} \nabla_{S}\bar{Y} & \partial_{\gamma }\bar{Y} & \partial_{\kappa }\bar{Y}\\ \nabla_{S}R^{\left(\varepsilon \right)} & \partial_{\gamma }R^{\left(\varepsilon \right)} & \partial_{\kappa }R^{\left(\varepsilon \right)}\\ \nabla_{S}h & \partial_{\gamma }h & \partial_{\kappa }h \end{array}]|}_{x\;=\;x_{0}}$$ (14)

and the system is solved:

$$x=x_{0}-B^{-1}\;b(x_{0})$$ (15)

These two operations are performed iteratively until the vectorial function is sufficiently close to zero. This is modeled by checking two quantities, which must be smaller than their respective tolerances: the residual on the whole vectorial function, with a higher tolerance:

$$res(\sigma ,\gamma ,\kappa )=\sqrt{{\bar{Y}}^{2}(\sigma ,\gamma ,\kappa )+\|R^{\left(\varepsilon \right)}(\sigma ,\gamma ,\kappa ){\|}^{2}+h^{2}(\sigma ,\gamma ,\kappa )}<tol_{1}$$ (16)

and the residual on the generalized yield function, with a lower tolerance:

$$\left|\bar{Y}(\sigma ,\gamma ,\kappa )\right|<tol_{2}$$ (17)

For the purposes of this work, the tolerances were set to $tol_{1}=10^{-6}$ and $tol_{2}=10^{-8}$.

### Model calibration

**Fig 1:**
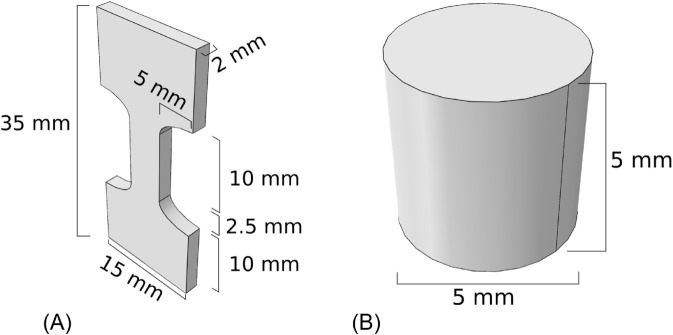
Calibration tests. Measures of the adopted geometries. (A) Dumbbell. (B) Cylindrical samples.

Aiming to calibrate the model, the experimental results reported by [29] were selected for comparison. They include monotonic uniaxial tests in tension and compression on femoral bovine cortical bone, performed on dumbbell-shaped and cylindrical-shaped specimens, respectively. Fig 1 reports the specimens dimensions and shape imported in the finite element environment Abaqus 2023 (Dassault Systèmes Simulia Corp., Providence, RI), to simulate the same experimental tests. Symmetry boundary conditions were assigned and imposed displacement was applied on the top face, with amplitudes of 0.17mm for tension and 0.1mm for compression. The total time period for the analysis was set to 1s, while the time parameter Δt in the model coincided with the increment size in the analysis, namely Δt=0.01s.

Furthermore, following the idea of a unique description suggested by [7], the considered model is applied also to experimental data concerning trabecular bone, namely the uniaxial tension and compression curves collected by [30]. As before, cylindrical specimens extracted from cadaveric lumbar spine trabecular bone were reproduced in Abaqus (diameter: 8mm, height: 20mm), with an applied displacement of 0.4mm in tension and compression.

**Fig 2:**
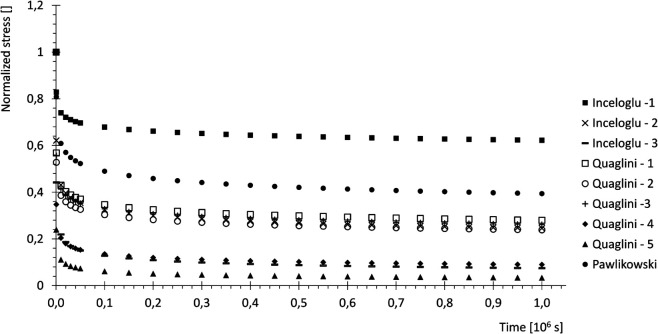
Relaxation curves. Experimental stress relaxation data from [31–33], fitted with power law and prolonged.

Regarding the viscous effects, most of the experimental relaxation data available in the literature were obtained within the elastic range, complicating the task of calibrating the viscous component of the viscoplastic model. To overcome this limitation, some assumptions were made: firstly, the experimental stress-strain curves used for calibration were assumed to be obtained from instantaneous loading, with no viscous effects present, and were therefore interpreted as limit curves that cannot be exceeded by the over-stresses developing during actual viscoplastic evolution. Secondly, the experimental data for relaxation reported by [31–33] were used to estimate the percentage of stress reduction at equilibrium. To this aim, these experimental curves were fitted with a power law and extended for a total time of $10^{6}\;s$, as in Fig 2, in order to reach their asymptotic values (percentages reported in Table 1).

**Table 1:** Percentage stress reduction from experimental relaxation curves.

Curve	Reduction	Curve	Reduction	Curve	Reduction
Q1	27%	Q4	9%	I3	62%
Q2	23%	Q5	3%	I2	25%
Q3	24%	P	39%	I3	7%

### Orthotropic axes association using the octree data structure

Tomography was used to detect the orthotropic axes within the bone geometry, in agreement with [16], where the voxel position vectors were associated with the three material axes.

Using this methodology it is possible to find a points cloud of material axes ${\mathbb{R}}^{a}$ that has to be correctly associated with the mesh discretization on a Finite Element Model. For each integration point in the mesh ${\mathbb{R}}^{ip}$ a point into ${\mathbb{R}}^{a}$ has to be found with minimal distance, to define the orthotropy directions and this means ${\mathbb{R}}^{ip}\times {\mathbb{R}}^{a}$ searches (see Eq 18).

$$\left\{x^{ip}\in {\mathbb{R}}^{ip}|x\in {\mathbb{R}}^{a}|\sqrt{\left((x^{ip}-x)\cdot (x^{ip}-x)\right)}=min\right\}$$ (18)

**Fig 3:**
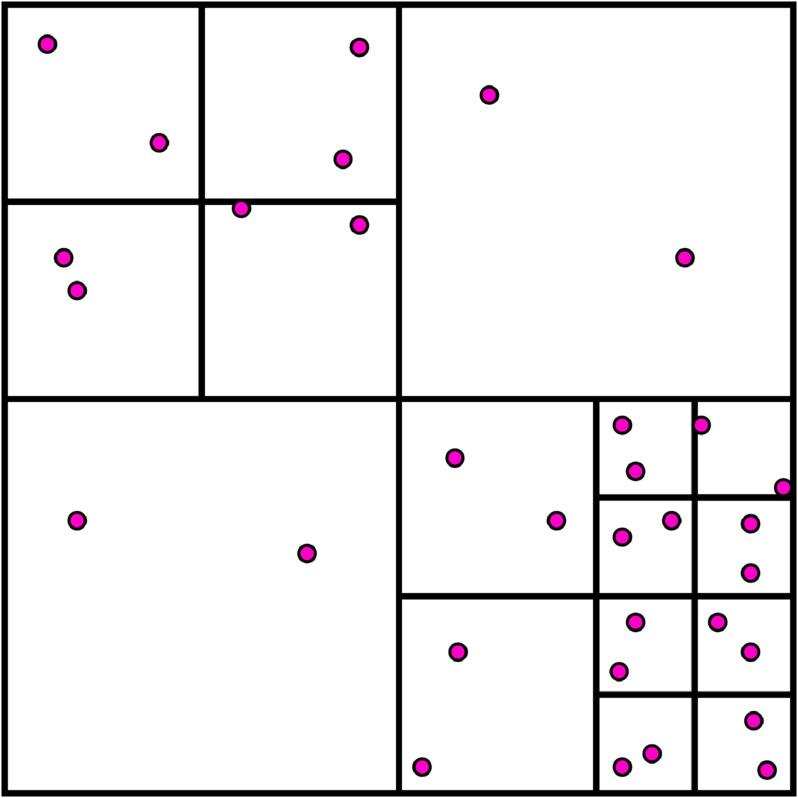
Two-dimensional example. A quadtree with a fixed number of points in each subspace equal to two.

Normally, the tomography scan produces a very dense points cloud, thus, to minimize computational cost, a more optimized procedure to define the material axes in an integration point consists in the octree method. It is a hierarchical data structure for spatial subdivision, where a cube space that includes the ${\mathbb{R}}^{a}$ points is divided into eight congruent cubes (subspace), each of which is split recursively until each minimal cube contains a fixed number of points (see the example in Fig 3).

The octree method has already been implemented in [34] where the comparisons with the sequential method using different numbers of points have been reported and it can be seen that, by increasing the problem size, the octree method significantly reduces the search time.

**Fig 4:**
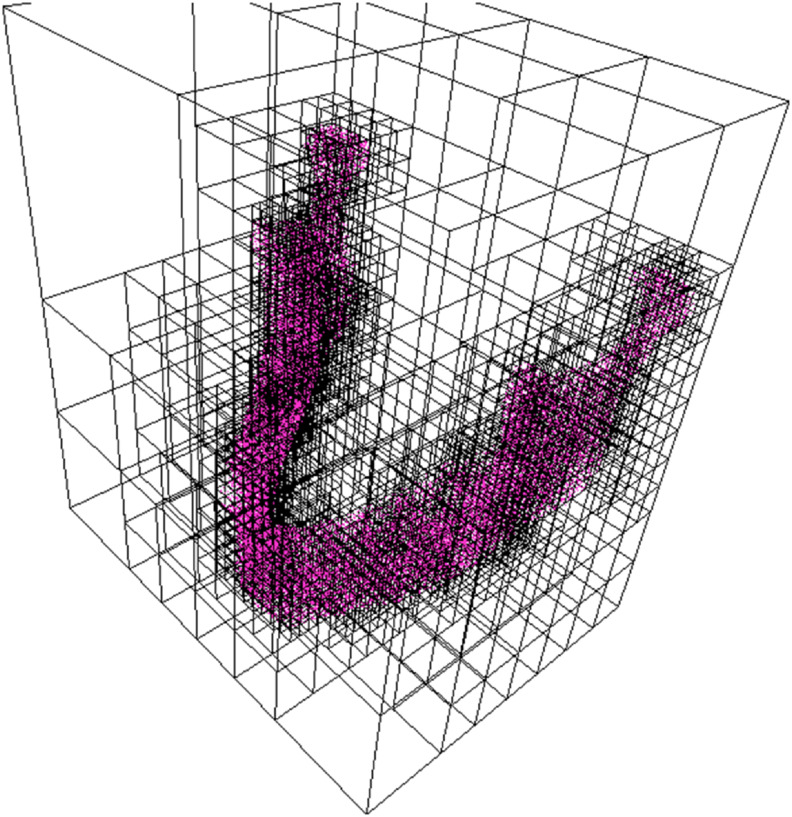
Octree spatial visualization.

Thus, by adopting this procedure, material heterogeneity was incorporated within the orthotropic constitutive model, which was calibrated on the basis of orthotropic elastic–plastic behavior, with the axes of orthotropy aligned according to the spatial variation of trabecular and cortical structures across different bone regions. In this way, the influence of microstructural organization was implicitly embedded within the constitutive description. A three-dimensional example of mandible point cloud obtained via tomography, and processed via the octree method, is reported in Fig 4.

## Results

### Calibration tests

**Fig 5:**
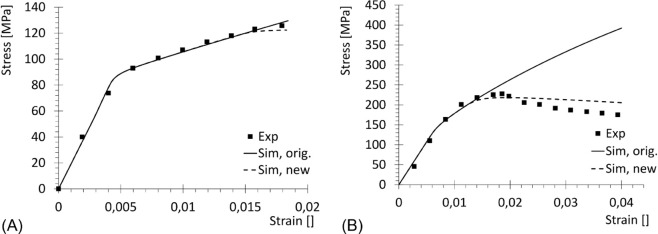
Uniaxial curves for cortical bone. Experimental (Exp) reported by [29], simulated with original hardening function (Eq 8) (Sim, orig.) and with new hardening-softening function (Eq 9) (Sim, new). (A) Tension. (B) Compression.

Material parameters were varied to obtain the best fit of the experimental curves obtained from [29] and [30]. The results are shown in Fig 5, which in particular highlights the improvement of the new hardening-softening function in mimicking the experimental results (Eq 9, dotted line in Fig 5) instead of the original one (Eq 8), while keeping fixed the other quantities. Calibrated numerical values for the involved parameters are collected in Tables 2–4 for the cortical bone.

**Table 2:** Elastic parameters for cortical bone, resulting from calibration.

Parameter	Value
*E*_1_ (GPa)	19.9
*E*_2_ (GPa)	11.2
*E*_3_ (GPa)	11.2
$\nu_{12}$	0.3
$\nu_{23}$	0.4
$\nu_{31}$	0.3
*G*_12_ (GPa)	5.7
*G*_23_ (GPa)	4.3
*G*_31_ (GPa)	5.7

**Table 3:** Damage, hardening and viscosity parameters for cortical bone, resulting from calibration.

Parameter	Value
*k*_*p*_	5
*k*_0_	0.0001
*y*_*r*_	5
*k*_*s*_	0.01
*s*_*h*_	10.5
*s*_*s*_	1.5
*m*	5
η(s)	0.01

**Table 4:** Yield parameters for cortical bone, resulting from calibration.

Parameter	Value
$\epsilon_{0}^{+}$	0.0018
$\epsilon_{0}^{-}$	0.015
$\xi_{0}$	0.25
$m_{1}$	(1, 0, 0)
$m_{2}$	(0, 1, 0)
$m_{3}$	(0, 0, 1)

**Fig 6:**
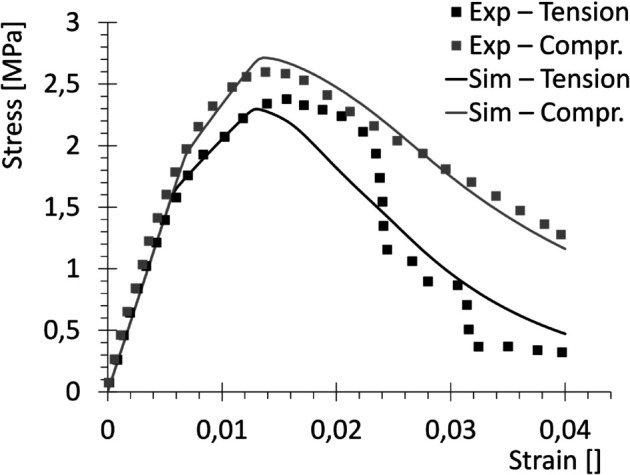
Uniaxial curves in tension and compression for trabecular bone. Comparison between experimental results by [30] (Exp) and numerical results obtained with the implemented model (Sim).

Trabecular bone was also evaluated, and results are reported in Fig 6, where dotted lines refer to experimental values, while continuous lines stand for numerical simulations. The calibrated numerical values for the parameters of trabecular bone are collected in Tables 5–7.

**Table 5:** Elastic parameters for trabecular bone, resulting from calibration.

Parameter	Value
*E*_1_ (MPa)	280
*E*_2_ (MPa)	160
*E*_3_ (MPa)	160
$\nu_{12}$	0.22
$\nu_{23}$	0.48
$\nu_{31}$	0.22
*G*_12_ (MPa)	80
*G*_23_ (MPa)	60
*G*_31_ (MPa)	80

**Table 6:** Damage, hardening and viscosity parameters for trabecular bone, resulting from calibration.

Parameter	Value
*k*_*p*_	5
*k*_0_	0.0001
*y*_*r*_	5.5
*k*_*s*_	0.005
*s*_*h*_	20
*s*_*s*_	4.5
*m*	5
η(s)	0.01

**Table 7:** Yield parameters for trabecular bone, resulting from calibration.

Parameter	Value
$\epsilon_{0}^{+}$	0.005
$\epsilon_{0}^{-}$	0.0075
$\xi_{0}$	0.25
$m_{1}$	(1, 0, 0)
$m_{2}$	(0, 1, 0)
$m_{3}$	(0, 0, 1)

When dealing with viscoplasticity, from Table 1 the average value of stress reduction equal to 24% was calculated and used to reduce the yield-deformation parameters obtained from calibration (Table 8), in order to estimate the equilibrium curve as obtained from infinitely slow loading, with no viscous effects. The resulting set of parameters has been therefore used for a proper simulation of viscoplastic effects, in the application of the model described in the following sections.

**Table 8:** Reduced yield-deformation parameters.

Parameter	$\epsilon_{0}^{+}$	$\epsilon_{0}^{-}$
Cortical bone	0.00044	0.0036
Trabecular bone	0.0012	0.0018

### Mesh sensitivity tests

The orthotropic axes assignment method allows to optimally orient the elements of a mesh with a given refinement, based on experimental data. The potential of this procedure is probed by testing it on two complex geometries obtained from CT scans, namely a mandible and a femur. For each geometry, four meshes of linear tetrahedral elements were built with increasing refinements, as reported in Table 9, and the above described procedure was applied to assign local orthotropic axes on each mesh.

**Table 9:** Mesh details (number of elements *n*_*e*_, number of nodes *n*_*n*_) for mandible and femur.

Mesh	$n_{e}$ mandible	$n_{n}$ mandible	$n_{e}$ femur	$n_{n}$ femur
1	51369	11910	48831	10752
2	113348	24012	219317	44080
3	487939	94779	475276	91868
4	2582404	476225	1453954	270117

**Fig 7:**
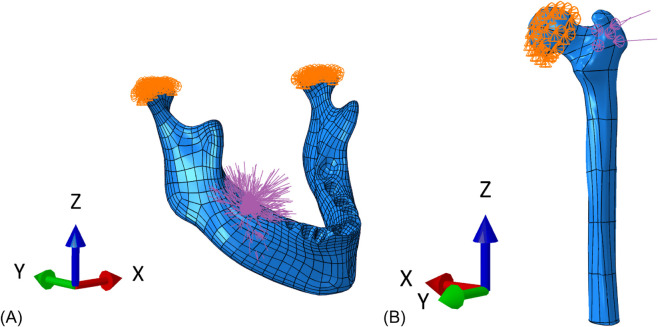
Loading and boundary conditions for the two geometries. (A) Mandible. (B) Femur.

As a preliminary test on these locally oriented geometries, a purely elastic material was assigned, using the transverse isotropic elastic properties obtained from cortical bone calibration; loads and boundary conditions were applied, as reported in Fig 7, and the resulting simulations were performed, with the aim of observing the effect on the predicted mechanical behavior of the increasingly accurate material orientation, resulting from higher refinements.

**Fig 8:**
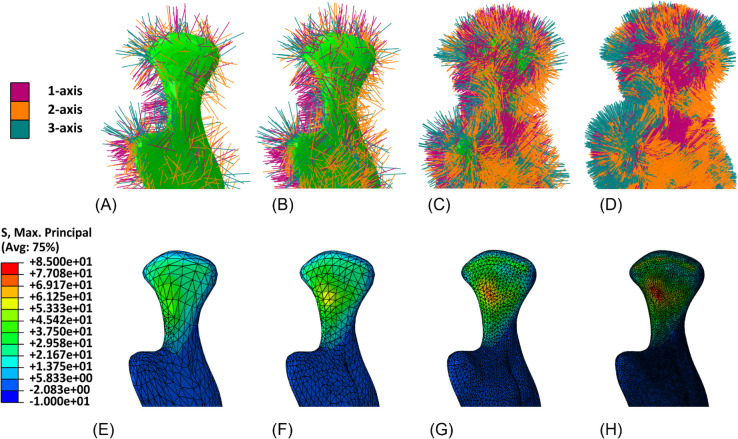
Mandible detail (left condyle). Assigned orthotropic axes and maximum principal stresses (*MPa*) for meshes with increasing refinement. (A) Mesh 1, with assigned orthotropic axes. (B) Mesh 2, with assigned orthotropic axes. (C) Mesh 3, with assigned orthotropic axes. (D) Mesh 4, with assigned orthotropic axes. (E) Mesh 1, maximum principal stresses. (F) Mesh 2, maximum principal stresses. (G) Mesh 3, maximum principal stresses. (H) Mesh 4, maximum principal stresses.

**Fig 9:**
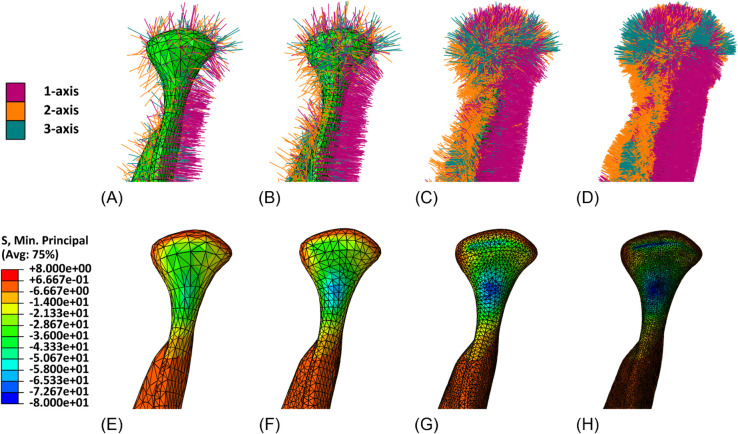
Mandible detail (left condyle). Assigned orthotropic axes and minimum principal stresses (*MPa*) for meshes with increasing refinement. (A) Mesh 1, with assigned orthotropic axes. (B) Mesh 2, with assigned orthotropic axes. (C) Mesh 3, with assigned orthotropic axes. (D) Mesh 4, with assigned orthotropic axes. (E) Mesh 1, minimum principal stresses. (F) Mesh 2,minimum principal stresses. (G) Mesh 3, minimum principal stresses. (H) Mesh 4, minimum principal stresses.

**Fig 10:**
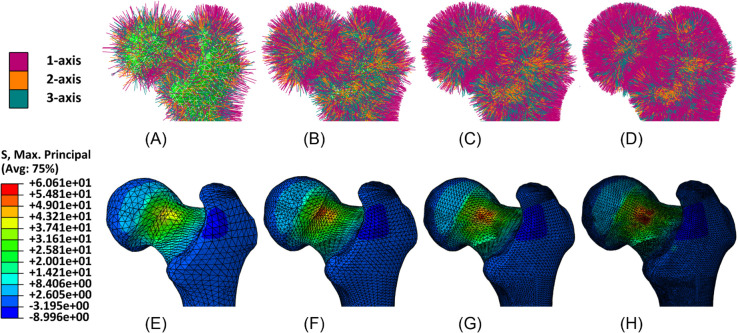
Femur detail. Assigned orthotropic axes and maximum principal stresses (*MPa*) for meshes with increasing refinement. (A) Mesh 1, with assigned orthotropic axes. (B) Mesh 2, with assigned orthotropic axes. (C) Mesh 3, with assigned orthotropic axes. (D) Mesh 4, with assigned orthotropic axes. (E) Mesh 1, maximum principal stresses. (F) Mesh 2, maximum principal stresses. (G) Mesh 3, maximum principal stresses. (H) Mesh 4, maximum principal stresses.

**Fig 11:**
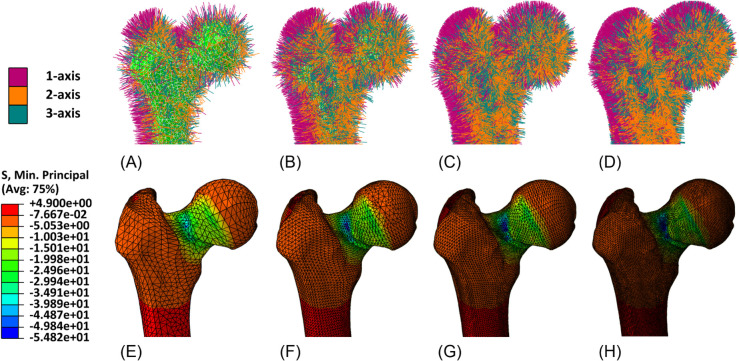
Femur detail. Assigned orthotropic axes and minimum principal stresses (*MPa*) for meshes with increasing refinement. (A) Mesh 1, with assigned orthotropic axes. (B) Mesh 2, with assigned orthotropic axes. (C) Mesh 3, with assigned orthotropic axes. (D) Mesh 4, with assigned orthotropic axes. (E) Mesh 1, minimum principal stresses. (F) Mesh 2,minimum principal stresses. (G) Mesh 3, minimum principal stresses. (H) Mesh 4, minimum principal stresses.

Qualitative results are reported in Figs 8–11, showing for both geometries the assigned orthotropic axes and the corresponding solutions in terms of maximum and minimum principal stresses, in regions where peaks are detected.

**Fig 12:**
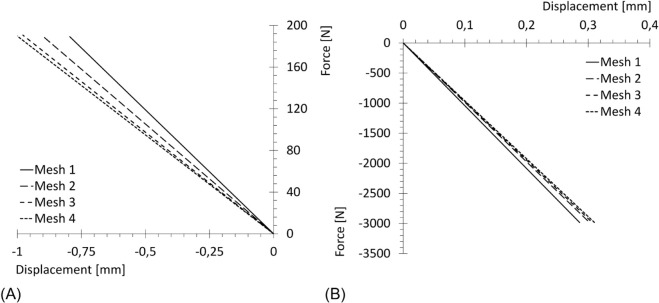
Force - displacement diagrams for elastic material. Force: sum on the constrained set of nodes of a relevant component of the reaction forces; displacement: same component of displacement in the test-node. (A) Mandible (relevant component: 3*^rd^*). (B) Femur (relevant component: 1*^st^*).

As a quantitative comparison, force - displacement plots were built for each mesh refinement and for both geometries. Force is computed as the sum on the constrained set of nodes (mandible condyles, femur head) of a representative component of the reaction forces (the 3*^rd^* component for the mandible, the 1*^st^* one for the femur), while the same component is measured for the displacement in a test-node chosen in the vicinity of displacement peaks. The results are reported in Fig 12.

**Fig 13:**
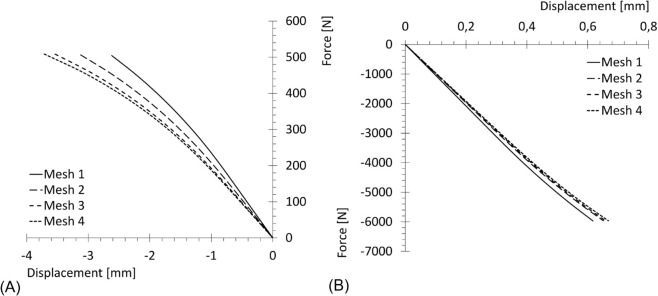
Force - displacement diagrams for complete material. Force: sum on the constrained set of nodes of a relevant component of the reaction forces; displacement: same component of displacement in the test-node. (A) Mandible (relevant component: 3*^rd^*). (B) Femur (relevant component: 1*^st^*).

Finally, the same tests were carried out using the complete implemented model for cortical bone, adapting the amplitude of the loads to ensure plasticity was reached. The resulting force - displacement plots are reported in Fig 13.

From Figs 12 and 13, it can be observed that Meshes 3 and 4 (the finest discretization) yield very similar results, indicating higher accuracy compared to the coarser meshes. However, increasing the number of elements also leads to a higher computational cost. Therefore, Mesh 3 can be regarded as providing a sufficiently accurate solution while maintaining a reasonable computational effort. Consequently, the discretization provided by Mesh 3 was selected as the most suitable for the following case study.

### Application: Dental implant

The potential of the entire procedure described in the present work was finally expressed in a practical application, namely the investigation of press fit action of a dental implant in bone tissue. The geometry consists of a portion of inferior human mandible obtained from computerized tomography, subdivided in a trabecular core region and a cortical shell, both modeled by adopting the calibrated parameters obtained in the previous section. It includes two cylindrical holes, of about 6mm of depth and 3.7mm diameter, corresponding to the preparation of the anatomical sites for the subsequent insertion of the implant.

In order to spare computational effort, the strategy suggested by [35] is applied. Instead of modeling the implant as an external object interacting with mandible sites, the press fit action is represented by means of a specific displacement field imposed to the nodes of the hole surfaces, representing the effect on the sites of the external surface of the implant. More specifically, the latter is assumed to be threaded, therefore a larger radial displacement linearly varying from 0.05mm to 0.15mm is imposed on the nodes corresponding to the thread, while a constant radial displacement of 0.05mm is imposed on the remaining nodes of the hole surfaces. The thread has a stress relaxation height of 0.4mm, while the lead measures 1.5mm. Boundary conditions are then completed by pinning the lateral faces of the mandible portion. A variable time step is selected, varying from 10s to 500s, in order to have a deeper insight about the initial loading process while limiting the computational cost in the subsequent part of the analysis. The displacement field is gradually imposed and then subsequently kept constant for a total observation time of $10^{4}\;s$.

**Fig 14:**
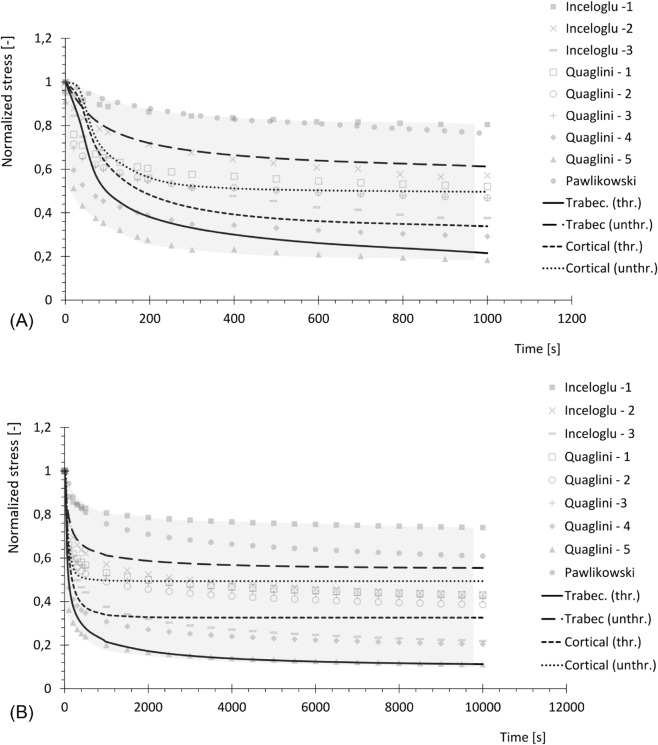
Normalized stress relaxation for cortical and trabecular bone tissue. Threaded (thr.) and unthreaded (unthr.) regions, obtained from simulation, in comparison with experimental results from literature. (A) Total time $10^{3}\;s$. (B) Total time $10^{4}\;s$.

Stress relaxation for cortical and trabecular bone tissues is shown in Fig 14, with normalized hydrostatic stress values from nodes on both threaded and unthreaded regions. Fig 14A focuses on a time range of 1000s, in order to compare the normalized simulated relaxation curve with the experimental one reported by [31,32] and [33]. Fig 14B extends the time range to $10^{4}\;s$, in order to observe the complete evolution of the viscoplastic effects.

**Fig 15:**
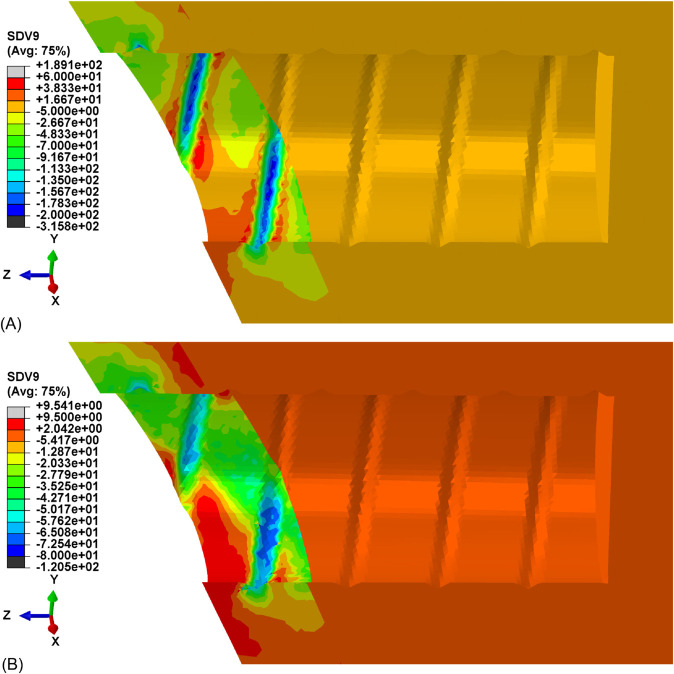
Hydrostatic stress distribution for cortical bone tissue. Contour map at the beginning of relaxation process. (A) Time = 0s. (B) Time = $10^{3}\;s$.

**Fig 16:**
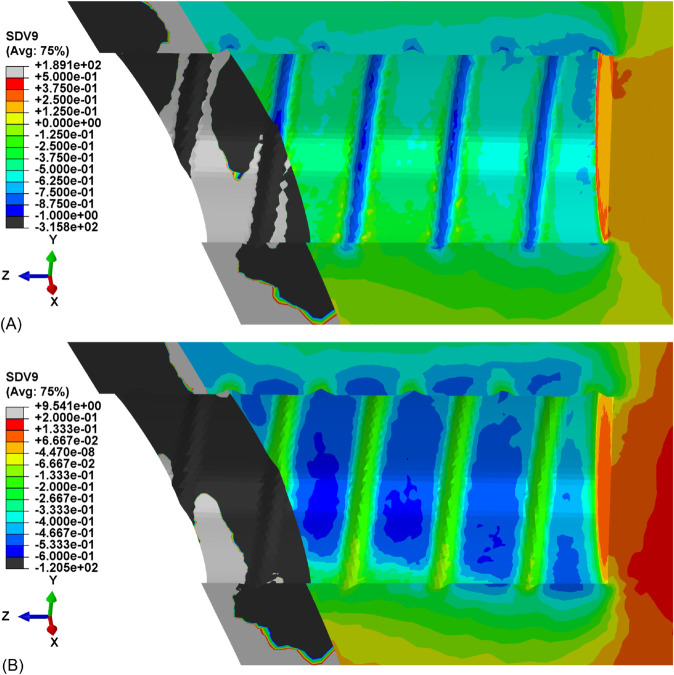
Hydrostatic stress distribution for trabecular bone tissue. Contour map at the beginning of relaxation process. (A) Time = 0s. (B) Time = $10^{3}\;s$.

Hydrostatic stress contour maps are reported in Fig 15 for cortical bone and in Fig 16 for trabecular bone, both comparing the stress distribution at the beginning of the relaxation process and after $10^{3}\;s$.

**Fig 17:**
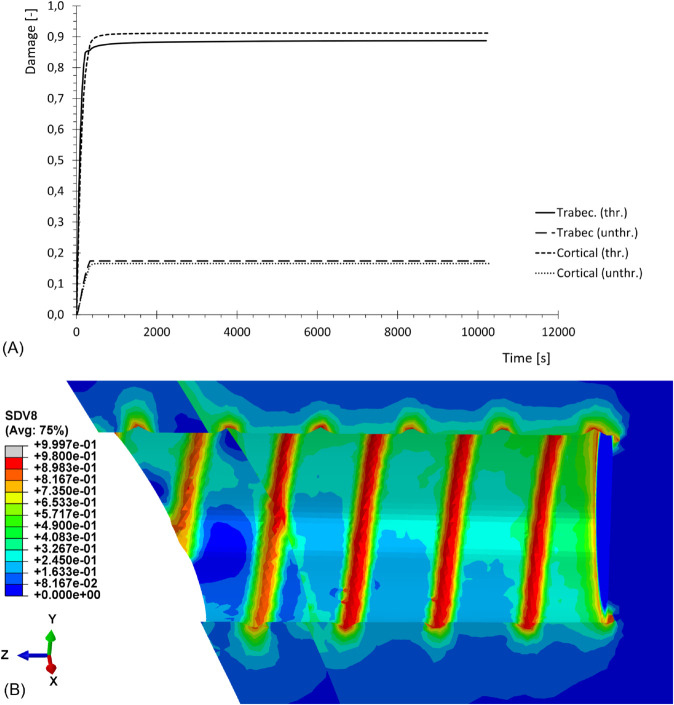
Damage during relaxation process. (A) Damage evolution for cortical and trabecular bone tissue, in threaded (thr.) and unthreaded (unthr.) regions, obtained from simulation. (B) Damage contour map after a relaxation time of $10^{3}\;s$.

Damage evolution for trabecular and cortical bone tissue is reported in Fig 17A, with damage values extracted from nodes on both threaded and unthreaded regions. The contour map in Fig 17B shows damage distribution over the dental site after 1000s of relaxation.

## Discussion

In the literature, several studies extended or adapted the model proposed by [8] with similar modeling philosophies to address specific applications or scales. For example, Werner et al. [37] employed a micro-FE approach based on voxelized *μ*-CT images of trabecular bone to investigate post-yield behavior of trabecular bone under multiaxial loading and large deformations, incorporating local elasto-plasticity and damage but omitting viscoplasticity. Similarly, Ovesy et al. [38] applied an explicit FE model using an anisotropic elasto-plastic formulation to simulate press-fit dental implant insertion, demonstrating the relevance of directional yield surfaces akin to those in Schwiedrzik’s work. In contrast, Lee et al. [39] proposed a purely phenomenological isotropic model capturing softening, plateau, and densification phases in trabecular bone, trading microstructural realism for computational efficiency. Akhlaghi et al. [40] and Khorshidparast et al. [41] both focused on implant-related bone damage, the former using a nonlinear isotropic micro-FE model with damage under large deformation, and the latter combining in-vitro loading, *μ*-CT imaging, and Digital Volume Correlation to quantify experimentally observed strain and damage. These studies emphasized mechanical degradation but did not incorporate rate effects or anisotropy. Kraiem et al. [25] proposed a continuum constitutive law for cortical bone explicitly including elastic-viscoplastic behavior and damage implemented in a macroscale context with a scalar damage variable and isotropic assumptions.

Among all these works, the constitutive model here presented and implemented was enriched with both a hardening-softening function r(κ) (Eq 9) and the inclusion of the detection of the orthotropic axes from CT, thus highlighting interesting steps forward compared to the existing literature. The resulting orthotropic constitutive model, defined through the fabric tensor, is precisely intended to incorporate microstructural information. Indeed, the model is calibrated on the basis of orthotropic elastic–plastic behavior, with the axes of orthotropy aligned according to the spatial variation of the trabecular and cortical structure in different regions of bone, implicitly embedding bone microstructural organization in the constitutive description. Moreover, the proposed model has proven capable of properly describing tension and compression curves for both cortical and trabecular bone, confirming its considerable versatility. In particular, the implementation of a hardening-softening function (Eq 9) enhances the predicted plastic evolution, ensuring a higher accuracy of the model, both in mimicking the experimental tests, as well as in more complex applications such as the dental implant.

In addition, the obtained results for Young’s and shear moduli in cortical bone are in agreement with those reported by [3], thus confirming the efficacy of model calibration.

The mesh sensitivity tests performed on the mandible and on the femur provide a valuable insight into the relationship between a proper orthotropic axes assignation and the quality of simulated results. From qualitative tests it can be observed that by refining the mesh, and therefore by representing the local orientation of the material with greater accuracy, peaks of maximum and minimum principal stresses become more visible, while they appear to be smoothed when orientation assignment is too rough. A more general and quantitative perspective is then outlined by the force-displacement plots, which account for the whole geometry. They clearly show the amount of under- or overestimation which is introduced by an excessively rough meshing and are a valuable tool to identify the optimal refinement, beyond which computational cost increases without leading to any further improvement.

The dental implant reconstruction allows to predict the complex nonlinear behavior of bone tissue in a realistic case [35]. The insertion of the implant in the dental site, represented by the threaded displacement field, causes the accumulation of stresses in correspondence with the threaded regions, as shown in Figs 15 and 16. The yield threshold is exceeded and, according to the model, over-stresses are accumulated, and then gradually released after full application of the load. This relaxation process, caused by the viscous properties of the model, is clearly visible in Fig 14A and 14B. The former confirms the good agreement between the range of experimental data and the simulated relaxation curves, for both cortical and trabecular bone material and at different points of the geometry, when considering a limited time range ($10^{3}\;s)$. The latter extends the predicted results to a wider time range ($10^{4}\;s$), which is sufficient for simulated curves to reach their asymptotic values. After complete relaxation, when over-stresses are completely vanished, some of the predicted residual stresses are still substantial. For example, for cortical bone in correspondence with the threaded region, the stress level is reduced to 33% of its initial value, which corresponds to about 64 MPa. This suggests that, even though correctly representing the relaxation of stresses associated to plastic regime, the model is not capable of predicting the exhaustion of over-stresses associated to viscoelasticity. The latter is, in fact, not included in the constitutive model, whose predictivity is therefore restricted to limited time ranges (about $10^{3}\;s$).

Damage evolution is also properly described by the model, with damage peaks accumulating in correspondence with the threaded regions, following the natural geometric pattern of the implant as shown in Fig 17B. Even though anisotropic damage laws are often introduced in the literature to capture the directional nature of material degradation, [36] in the present work, the anisotropic nonlinear response has been primarily assigned to the plasticity model, while adopting a simplified scalar damage evolution law, that can be readily characterized by means of standard uniaxial loading–unloading experiments, ensuring tractability and robustness of the model calibration. Most of the damage accumulation occurs during the loading process, but a small variation is still visible during the relaxation process: due to the viscoplastic evolution, a small amount of unrecoverable strains is still accumulating, and this is related to the physical formation of microcracks and the consequent accumulation of damage, even though in small percentages. Damage values at the end of the process are considerably high, which is consistent with the absence, in the model, of any bone remodeling process. Physical bone tissue, in fact, has a self-repairing ability which allows, through biological mechanisms, to counteract the accumulation of microcracks, by removing the damaged material and replacing it with newly produced tissue.

The selected constitutive model therefore has a limited capacity of predicting the correct amount of damage accumulation on long time ranges, during which bone self-repairing ability would be effective. However, it is completely functional in limited time ranges, describing in a realistic way damage evolution and its distribution along the geometrical patterns of the specific bone under analysis. Moreover, it must be highlighted that, since the reference experimental data are restricted to uniaxial tests [7,29,30], they are limited to provide an exhaustive description of all the material parameters, which therefore represent plausible values, aimed at providing possible applications of the model. For a better parameter identification, a wider range of bone-specific experiments would be required. This is particularly true for the viscous part of the problem, whose calibration has been significantly affected by the lack of specific and systematic experimental data, especially in plastic regime [31–33]. The assumptions adopted in this work allowed to compensate for this difficulty with reasonable approximation, leading to plausible predicted values [37].

## Conclusion

The work presented efficient tools for bone reconstruction and modeling, combining the predictivity of a complex constitutive model with the geometrical accuracy offered by CT scans, and suggesting an efficient method for assigning material orientation from experimental data.

The comparison with experimental data for both cortical and trabecular bone confirmed the flexibility of the model, which can adapt to different types of bone tissue simply by modifying the proper set of values for the material parameters.

Mesh sensitivity tests, efficiently performed through an octree search algorithm, highlighted the importance of an accurate assignment of the material orientation, which effectively influences the precision of the simulated patterns and must be therefore taken into account when choosing the optimal mesh refinement, contrary to what is still commonly adopted in the literature. The application of the overall procedure to the realistic case of the dental implant outlined the potential of the presented procedure. Further improvements will focus on coupling the softening evolution with damage, which would assure the same predictivity while better relating to the physical causes of such process as shown in [4].

However, the full potential of the model has been properly expressed within time ranges of about $10^{3}\;s$, with realistic predictions concerning both the stress distributions and their progressive relaxation, and the evolution of damage were fully consistent with the geometry of the problem.

To conclude, the procedure presented in this work offers a solid starting point for further developments, allowing to compare simulated results from sophisticated constitutive models with experimental data even on complex geometries, and thus appears as a robust and more reliable model that can be adapted in all those applications which require the description of both cortical and trabecular bone with an elasto-viscoplastic manner, from sport science and injuries protection, to surgical and dental implant evaluations.

## Supporting information

S1 File The raw/processed data required to reproduce these findings are available as supplementary material. (ODS)

## References

[1] Ritchie RO, Buehler MJ, Hansma P. Plasticity and toughness in bone. Physics Today. 2009;62(6):41–7. doi: 10.1063/1.3156332

[2] Pietruszczak S, Inglis D, Pande GN. A fabric-dependent fracture criterion for bone. J Biomech. 1999;32(10):1071–9. doi: 10.1016/s0021-9290(99)00096-2 10476845

[3] Mirzaali MJ, Schwiedrzik JJ, Thaiwichai S, Best JP, Michler J, Zysset PK, et al. Mechanical properties of cortical bone and their relationships with age, gender, composition and microindentation properties in the elderly. Bone. 2016;93:196–211. doi: 10.1016/j.bone.2015.11.018 26656135

[4] Megías R, Vercher-Martínez A, Belda R, Peris JL, Larrainzar-Garijo R, Giner E, et al. Numerical modelling of cancellous bone damage using an orthotropic failure criterion and tissue elastic properties as a function of the mineral content and microporosity. Comput Methods Programs Biomed. 2022;219:106764. doi: 10.1016/j.cmpb.2022.106764 35366593

[5] Zysset PK, Curnier A. A 3D damage model for trabecular bone based on fabric tensors. J Biomech. 1996;29(12):1549–58. doi: 10.1016/s0021-9290(96)80006-6 8945653

[6] Zysset P, RincoÂ´n L. An alternative fabric-based yield and failure criterion for trabecular bone. Mechanics of biological tissue. 2006. p. 457–70.

[7] Garcia D, Zysset PK, Charlebois M, Curnier A. A three-dimensional elastic plastic damage constitutive law for bone tissue. Biomech Model Mechanobiol. 2009;8(2):149–65. doi: 10.1007/s10237-008-0125-2 18398628

[8] Schwiedrzik JJ, Zysset PK. An anisotropic elastic-viscoplastic damage model for bone tissue. Biomech Model Mechanobiol. 2013;12(2):201–13. doi: 10.1007/s10237-012-0392-9 22527365

[9] Charlebois M, Jirásek M, Zysset PK. A nonlocal constitutive model for trabecular bone softening in compression. Biomech Model Mechanobiol. 2010;9(5):597–611. doi: 10.1007/s10237-010-0200-3 20238139

[10] Zioupos P, Hansen U, Currey JD. Microcracking damage and the fracture process in relation to strain rate in human cortical bone tensile failure. J Biomech. 2008;41(14):2932–9. doi: 10.1016/j.jbiomech.2008.07.025 18786670

[11] Atsumi N, Tanaka E, Iwamoto M, Hirabayashi S. Constitutive modeling of cortical bone considering anisotropic inelasticity and damage evolution. Mech Eng J. 2017;4(4).

[12] Soni A, Negi A, Kumar S, Kumar N. An IGA based nonlocal gradient-enhanced damage model for failure analysis of cortical bone. Eng Fract Mech. 2021;255:107976.

[13] Natali AN, Carniel EL, Pavan PG. Constitutive modelling of inelastic behaviour of cortical bone. Med Eng Phys. 2008;30(7):905–12. doi: 10.1016/j.medengphy.2007.12.001 18207444

[14] Johnson TPM, Socrate S, Boyce MC. A viscoelastic, viscoplastic model of cortical bone valid at low and high strain rates. Acta Biomater. 2010;6(10):4073–80. doi: 10.1016/j.actbio.2010.04.017 20417735

[15] Pettermann HE, Reiter TJ, Rammerstorfer FG. Computational simulation of internal bone remodeling. ARCO. 1997;4(4):295–323. doi: 10.1007/bf02737117

[16] Toniolo I, Salmaso C, Bruno G, De Stefani A, Stefanini C, Gracco ALT, et al. Anisotropic computational modelling of bony structures from CT data: an almost automatic procedure. Comput Methods Programs Biomed. 2020;189:105319. doi: 10.1016/j.cmpb.2020.105319 31951872

[17] Toniolo I, Berardo A, Foletto M, Fiorillo C, Quero G, Perretta S, et al. Patient-specific stomach biomechanics before and after laparoscopic sleeve gastrectomy. Surg Endosc. 2022; 1–14. doi: 10.1007/s00464-022-09233-7 35451669 PMC9028903

[18] Knowles NK, Neeteson N, Boyd SK. High performance multi-platform computing for large-scale image-based finite element modeling of bone. Comput Methods Programs Biomed. 2022;225:107051. doi: 10.1016/j.cmpb.2022.107051 35939979

[19] Vollmer D, Meyer U, Joos U, Vègh A, Piffko J. Experimental and finite element study of a human mandible. J Craniomaxillofac Surg. 2000;28(2):91–6. doi: 10.1054/jcms.2000.0125 10958421

[20] Pisano AA, Fuschi P. Limit analysis of human proximal femur. J Mech Behav Biomed Mater. 2021;124:104844. doi: 10.1016/j.jmbbm.2021.104844 34601433

[21] Hellmich C, Kober C, Erdmann B. Micromechanics-based conversion of CT data into anisotropic elasticity tensors, applied to FE simulations of a mandible. Ann Biomed Eng. 2008;36:108–22. 17952601 10.1007/s10439-007-9393-8

[22] Blanchard R, Morin C, Malandrino A, Vella A, Sant Z, Hellmich C. Patient-specific fracture risk assessment of vertebrae: a multiscale approach coupling X-ray physics and continuum micromechanics. Int J Numer Method Biomed Eng. 2016;32(9):10.1002/cnm.2760. doi: 10.1002/cnm.2760 26666734

[23] Luisier B, Dall’Ara E, Pahr DH. Orthotropic HR-pQCT-based FE models improve strength predictions for stance but not for side-way fall loading compared to isotropic QCT-based FE models of human femurs. J Mech Behav Biomed Mater. 2014;32:287–99. doi: 10.1016/j.jmbbm.2014.01.006 24508715

[24] Lu Y, Maquer G, Museyko O, Püschel K, Engelke K, Zysset P, et al. Finite element analyses of human vertebral bodies embedded in polymethylmethalcrylate or loaded via the hyperelastic intervertebral disc models provide equivalent predictions of experimental strength. J Biomech. 2014;47(10):2512–6. doi: 10.1016/j.jbiomech.2014.04.015 24818795

[25] Kraiem T, Barkaoui A, Merzouki T, Chafra M. Computational approach of the cortical bone mechanical behavior based on an elastic viscoplastic damageable constitutive model. Int J Appl Mech. 2020;12(7).

[26] Dassault Systemes SIMULIA Corp. Providence, RI: Dassault Systemes; 2014.

[27] Perzyna P. Fundamental problems in viscoplasticity. Adv Appl Mech. 1966;9:243–377.

[28] Etse G, Carosio A. Constitutive equations and numerical approaches in rate dependent material formulations. Mecanica Computacional. 1999;5:289–96.

[29] Li S, Demirci E, Silberschmidt VV. Variability and anisotropy of mechanical behavior of cortical bone in tension and compression. J Mech Behav Biomed Mater. 2013;21:109–20. doi: 10.1016/j.jmbbm.2013.02.021 23563047

[30] Morgan EF, Unnikrisnan GU, Hussein AI. Bone mechanical properties in healthy and diseased states. Annu Rev Biomed Eng. 2018;20:119–43. doi: 10.1146/annurev-bioeng-062117-121139 29865872 PMC6053074

[31] Quaglini V, La Russa V, Corneo S. Nonlinear stress relaxation of trabecular bone. Mech Res Commun. 2009;36:275–83.

[32] Inceoglu S, Akbay A, McLain RF. Stress relaxation at the bone-pedicle screw interface in human bone. Spine (Phila Pa 1976). 2006;31(12):1321–6. doi: 10.1097/01.brs.0000218478.70656.63 16721293

[33] Pawlikowski M, Barcz K. Non-linear viscoelastic constitutive model for bovine cortical bone tissue. Biocybern Biomed Eng. 2016;36:491–8.

[34] Mazzucco G, Xotta G, Salomoni VA, Majorana C. Integral-type regularization of non associated softening plasticity for quasi brittle materials. Comput Struct. 2019;224:106120.

[35] Natali AN, Carniel EL, Pavan PG. Investigation of viscoelastoplastic response of bone tissue in oral implants press fit process. J Biomed Mater Res B Appl Biomater. 2009;91(2):868–75. doi: 10.1002/jbm.b.31469 19637368

[36] Pensée V, Kondo D, Dormieux L. Micromechanical analysis of anisotropic damage in brittle materials. J Eng Mech. 2002;128(8):889–97.

[37] Werner B, Ovesy M, Zysset PK. An explicit micro-FE approach to investigate the post-yield behaviour of trabecular bone under large deformations. Int J Numer Method Biomed Eng. 2019;35(5):e3188. doi: 10.1002/cnm.3188 30786166

[38] Ovesy M, Aeschlimann M, Zysset PK. Explicit finite element analysis can predict the mechanical response of conical implant press-fit in homogenized trabecular bone. J Biomech. 2020;107:109844. doi: 10.1016/j.jbiomech.2020.109844 32517857

[39] Lee C-S, Lee J-M, Youn B, Kim H-S, Shin JK, Goh TS, et al. A new constitutive model for simulation of softening, plateau, and densification phenomena for trabecular bone under compression. J Mech Behav Biomed Mater. 2017;65:213–23. doi: 10.1016/j.jmbbm.2016.08.028 27592290

[40] Akhlaghi P, Khorshidparast S, Rouhi G. Investigation on primary stability of dental implants through considering peri-implant bone damage, caused by small and large deformations: A validated non-linear micro finite element study. J Mech Behav Biomed Mater. 2023;146:106062. doi: 10.1016/j.jmbbm.2023.106062 37549522

[41] Khorshidparast S, Akhlaghi P, Rouhi G, Barikani H. Measurement of bone damage caused by quasi-static compressive loading-unloading to explore dental implants stability: Simultaneous use of in-vitro tests, *μ*-CT images, and digital volume correlation. J Mech Behav Biomed Mater. 2023;138:105566. doi: 10.1016/j.jmbbm.2022.105566 36435034

